# Supplementary material for: PopTargs: a database for studying population evolutionary genetics of human microRNA target sites
Source: Database (Oxford). 2019 Oct 11;2019:baz102. doi: 10.1093/database/baz102 (PMC6790967; doi:10.1093/database/baz102)
Supplement: Supplemtary_Table_1_baz102 [file supplemtary_table_1_baz102.pdf]

Supplementary Table 1. Accession number of sRNA-seq experiments processed in this study.

Tissue	EBI-ENA accession number
Blood	DRX012360; DRX012361; DRX012362; SRX318164; SRX318180; SRX386679; SRX386680; SRX386681; SRX426123; SRX426124; SRX426125; SRX426475; SRX426478; SRX666575; SRX666576; SRX666577; SRX666578; SRX666579
Brain	SRX182778; SRX375448; SRX375450; SRX375452; SRX375455; SRX375461; SRX375462; SRX375463; SRX375464; SRX375466; SRX375467
Breast	SRX513283; SRX513284; SRX513285; SRX513286
Cerebellum	SRX182779
Heart	SRX182780
Kidney	SRX182781
Liver	SRX353113
Lung	DRX003170; DRX003171
Placenta	SRX262196; SRX262197; SRX262198; SRX262199; SRX262200; SRX262201; SRX262202; SRX262203
Testis	SRX182782; SRX271415; SRX271416; SRX271417
